# Results of the Nerve Transfers and Secondary Procedures to Restore Shoulder and Elbow Function in Traumatic Upper Brachial Plexus Palsy

**DOI:** 10.3390/jcm13237396

**Published:** 2024-12-04

**Authors:** Piotr Czarnecki, Michał Górecki, Leszek Romanowski

**Affiliations:** Department of Traumatology, Orthopaedics and Hand Surgery, Poznan University of Medical Sciences, 61-545 Poznań, Poland; pczarnecki@orsk.pl (P.C.);

**Keywords:** nerve transfer, brachial plexus palsy, nerve reconstruction, restore shoulder/elbow function

## Abstract

**Background:** Damage to the upper trunk of the brachial plexus, often caused by high-energy trauma, leads to significant functional impairment of the upper limb. This injury primarily affects the C5 and C6 roots, resulting in paralysis of muscles critical for shoulder and elbow function. If spontaneous nerve regeneration does not occur within 3–6 months post-injury, surgical intervention, including nerve transfers, is recommended to restore function. **Methods:** This study evaluates long-term outcomes of nerve transfer surgeries performed between 2013 and 2023 on 16 adult patients with post-traumatic brachial plexus injuries. The most common cause of injury was motorcycle accidents. Nerve transfers targeted shoulder and elbow function restoration, including transfer of the accessory nerve to the suprascapular nerve, the radial nerve branch to the long or medial head of the triceps brachii to the axillary nerve, or the transfer of motor fascicles of the ulnar and median nerves (double Oberlin) to the brachialis and biceps brachii motor nerves. **Results:** Postoperative results showed varying degrees of functional recovery. In the shoulder, most patients achieved stabilization and partial restoration of active movement, with average flexion up to 92° and abduction up to 78°. In the elbow, full flexion with M4 strength was achieved in 64% of patients. In both the shoulder and the elbow, double nerve transfers yield better long-term outcomes than single transfers. Secondary procedures, such as tendon transfers, were required in some cases to improve limb strength. **Conclusions:** The study concludes that nerve transfers offer reliable outcomes in restoring upper limb function, although additional surgeries may be necessary in certain cases.

## 1. Introduction

Damage to the upper trunk of the brachial plexus or avulsion of the C5 and C6 root results in significant limitations of upper limb function and is most commonly the consequence of high-energy trauma, such as a motor vehicle accident, typically involving motorcycles. It leads to dysfunction of the suprascapular, subscapular, axillary, and musculocutaneous nerves. This results in paralysis of the rotator cuff muscles, deltoid muscle, biceps brachii, and brachialis muscles. Consequently, there is marked shoulder dysfunction with the absence of active abduction, flexion, and rotation of the arm, as well as a lack of active elbow flexion [1,2,3]. If spontaneous nerve regeneration does not occur, surgical intervention should be considered approximately 3–6 months post-injury to restore shoulder and elbow function. To identify the type and extent of nerve injury, imaging studies such as magnetic resonance imaging (MRI) and the assessment of motor fiber conduction, particularly motor evoked potential, may prove helpful [4,5,6]. Due to the complexity of diagnosing the injury, the need for a rapid clinical outcome, and the inability to repair certain types of damage (especially avulsion injuries), nerve transfer techniques should be considered [4,7]. This approach involves transferring a less critical nerve branch directly to the distal portion of the damaged nerve, bypassing the primary cause (e.g., avulsion). The short distance to the muscle offers the potential for rapid nerve regeneration and early restoration of function, and targeted neurotization results in a better outcome [8]. The most used donor nerves are the accessory nerve (AN), long thoracic nerve, branch of the radial nerve (RN) to the triceps brachii muscle, intercostal nerves, fascicles of the ulnar nerve (UB), and median nerve (MB) [9,10,11].

In specifically defined cases, nerve repair at the level of the brachial plexus (typically after direct trauma) is possible through direct repair and end-to-end suturing or reconstruction using a graft or end-to-side suturing [10,12,13]. In the case of delayed treatment of an inveterate brachial plexus injury, irreversible atrophy of the denervated muscles occurs, and then nerve transfers are not the treatment of choice. Secondary treatment consisting of non-anatomical tendon transfers or ultimately shoulder arthrodesis should be considered [3,14].

The aim of this study is to present long-term results after nerve transfers in the shoulder and/or elbow region performed for posttraumatic brachial plexus injury in adult and adolescent patients.

## 2. Materials and Methods

Between 2013 and 2023, a single surgeon operated on 20 patients aged 15 to 48 years (mean age 30 years) with predominantly partial brachial plexus injuries, primarily affecting the upper and middle trunks, for whom nerve transfers were indicated and feasible. Ultimately, the results of 16 of them were evaluated because the remaining 4 patients had too short an observation time or were lost in follow-up. The most common cause of injury was motorcycle accidents. Most patients presented with either no active shoulder movement or significantly limited movement. Additionally, 9 patients lacked active elbow flexion, and one patient had only isolated limitation of active elbow flexion.

In 13 patients, nerve transfers were performed to improve shoulder function, and in 4 cases, brachial plexus neurolysis was carried out. Furthermore, 11 patients underwent nerve transfers to restore elbow flexion.

We qualified patients for surgery based on the following criteria:

- Roots avulsion or upper/middle trunk injury in MRI and/or USG;
- No regeneration until 6 months;
- Regeneration excluded in nerve conduction studies;
- The full or near-full function of the radial nerve was tested clinically with triceps brachii strength of M4 or M5;
- Full function of median and ulnar nerves tested clinically;
- Patient compliant with physiotherapy.

**Figure 1:**
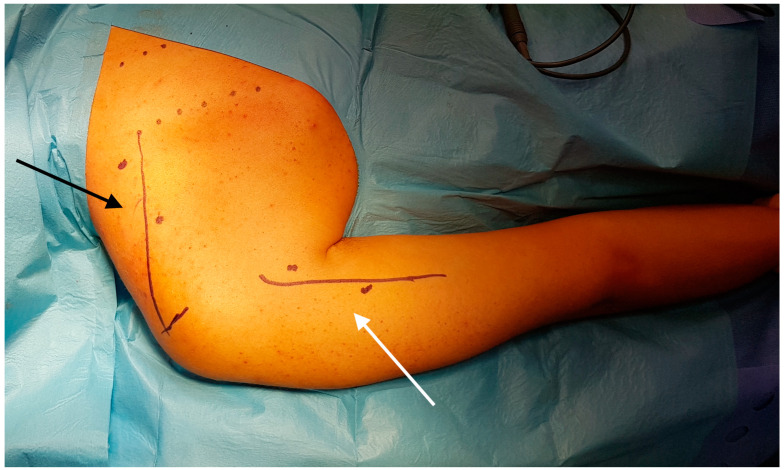
A prone position of the patient with marked anatomical landmarks for transferring the accessory nerve to the suprascapular nerve (black arrow) and the branch of the radial nerve for the triceps brachii to the axillary nerve (white arrow).

**Figure 2:**
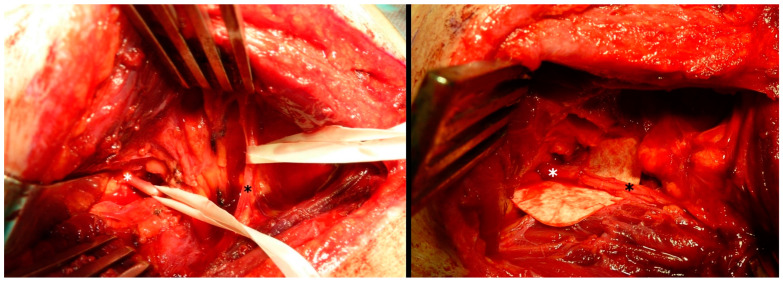
Intraoperative photo on the left side showing the prepared and protected accessory nerve (black asterisk) and the suprascapular nerve (white asterisk) and on the right side after the end-to-end transfer.

**Figure 3:**
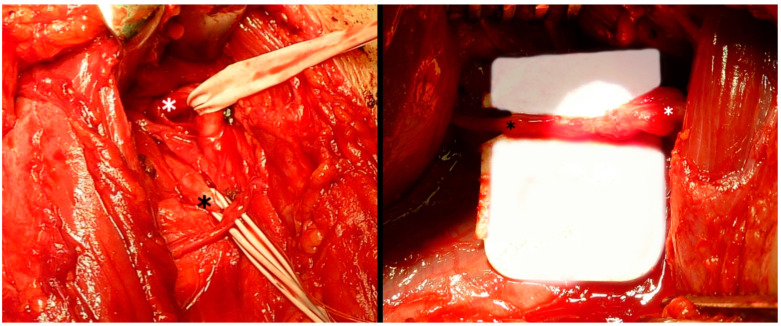
Intraoperative photo on the left side showing the prepared and protected axillary nerve (white asterisk) and the branch of the radial nerve of the triceps brachii (black asterisk) and on the right side after the end-to-end transfer.

**Figure 4:**
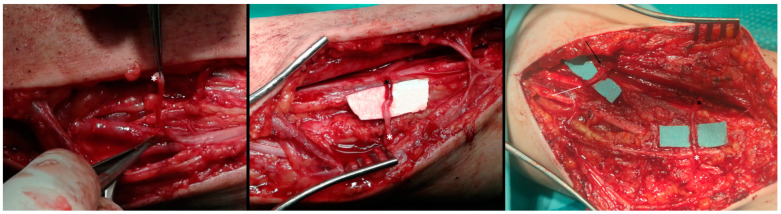
Intraoperative photo on the left side showing the dissected and isolated fascicle from the middle of the median nerve (white asterisk), in the middle photo after the Oberlin end-to-end transfer to the motor branch of the biceps brachii muscle (black asterisk), and on the right side after the double Oberlin transfer with the added transfer of the fascicle from the ulnar nerve (white arrow) to the motor branch of the brachialis muscle (black arrow).

Shoulder functions were restored by transfer of the accessory nerve to the suprascapular nerve (10 patients). In 11 patients, axillary nerve was neurotized using radial nerve branch to the long or medial head of triceps brachii as an isolated procedure or in combination with nerve transfer around shoulder described above. Elbow flexion was restored by transfer of ulnar and median nerve motor fascicles (double Oberlin) to the brachialis and biceps brachii motor nerves, respectively, in 8 patients. Three patients had only one transfer performed for elbow flexion, two using the ulnar nerve motor fascicle to the motor branch of the brachialis and one using the medial nerve fascicle to the motor branch of the biceps brachii. All nerve transfers were performed under an intraoperative microscope using end-to-end suture without tension (also in full passive limb range of motion) and without the need for a nerve graft. In most cases where both shoulder and elbow nerve transfers were performed, they were performed in two surgeries 1–2 months apart (except for one case where the transfers were performed in one surgery) [Figure 1, Figure 2, Figure 3 and Figure 4].

All nerve transfers and the postoperative protocol used were performed based on the surgical technique described in detail in Mackinnon et al. [15].

In some patients, tendon transfers were also performed to improve strength and function of the shoulder or elbow. Each patient underwent between 1 and 5 operations, with an average of just over 2 surgeries [Table 1].

Table 2 provides detailed information on the types of transfers performed, categorized by shoulder and elbow [Table 2].

The average time from injury to the first surgery was slightly over 6 months. The follow-up period was at least 12 months, with an average of just above 3 years. During this time, patients underwent individual electrostimulation of the affected muscles and were rehabilitated under the supervision of a physiotherapist. We assessed the improvement in limb function by evaluating the range of active motion and muscle strength using the British Medical Research Council (MRC) scale.

## 3. Results

The shoulder transfers resulted in stabilization of the glenohumeral joint in all patients, and in 11 patients, they provided active movement with varying degrees of flexion, abduction, and external rotation. Flexion and/or abduction above 90° was achieved in eight patients, while three patients had less than 90°, and two patients had no active movement. The average active range of motion was flexion 103°, abduction 83°, and external rotation 30°, with seven patients achieving muscle strength M4, four patients M3, and two patients M1.

The elbow transfers resulted in full flexion with strength M4 or greater in seven patients, flexion in the range of 0–90° with strength M3 in three patients, and no active flexion in one patient. No motor deficits were observed in the median and ulnar nerves after graft harvesting; however, one patient experienced temporary sensory weakness in the ulnar nerve distribution. Additionally, five patients underwent secondary procedures, such as latissimus dorsi transfer (one patient), Steindler’s flexor transfer (two patients), and trapezius transfer to the deltoid (one patient), which were performed to improve limb strength in most cases from M3 to M3+ or M4. All results presented include additional tendon transfers in the affected patients [Table 3].

Despite the small size of the groups, it is worth noting the differences in the range of motion and muscle strength depending on the variables:

(1) Patients who underwent surgery within 6 months of the injury achieved a better range of motion than those operated on after 6 months (but no later than 12 months), with flexion and abduction ranges of 106 degrees and 95 degrees, respectively, compared to 96 degrees and 65 degrees.
(2) Patients who underwent a single nerve transfer in the shoulder (radial branch to axillary—five patients, included suprascapular nerve decompression in two patients) demonstrated the same average range of motion as patients after double nerve transfers (eight patients) by 103 degrees of flexion and 83 degrees of abduction, but the average range of motion for patients with clear single transfer (without suprascapular nerve neurolysis) was much worse and amounted to 73 degrees of flexion and 57 degrees of abduction.
(3) A higher percentage of patients with M4 or greater outcomes (75%) is achieved following double Oberlin transfers compared to single Oberlin transfers (33%).
(4) Patients with high-energy trauma (motorcycle accident) achieved significantly worse results in the shoulder compared to patients with other lower-energy injuries. In the shoulder, the range of flexion was about 1.5 times smaller, and the range of abduction and external rotation was about 2 times smaller (83/57/20 vs. 119/106/41). Only 1/3 of patients achieved M4 strength or greater compared to the other patients, where 3/4 of the subjects achieved such strength. The results in the elbow were comparable, where about 2/3 of patients achieved functional movement and M4 strength or greater, while only in the group of other patients were M5 results noted, which were not achieved in the group with high-energy trauma.

## 4. Discussion

Upper brachial plexus injuries represent 15–20% of supraclavicular plexus injuries [16]. Repairing these injuries usually yields a favorable prognosis as hand function remains intact. In cases of root avulsions, where nerve repair is not feasible, nerve transfers provide significantly better outcomes compared to tendon or muscle transfers or shoulder arthrodesis. The primary goals of nerve transfers are restoring elbow flexion and shoulder stability and abduction [3,17].

While shoulder arthrodesis and muscle/tendon transfers provide limited results, nerve transfers offer better outcomes. For example, transferring the distal spinal accessory nerve to the suprascapular nerve can restore approximately 45° of shoulder abduction [18,19]. The reason for this poor outcome is probably that the number of myelinated fibers in the accessory nerve is insufficient to match the number of fibers in the suprascapular nerve (1300 vs. 3800, respectively). In addition, during the process of regeneration, the number of nerve fibers must be divided to innervate both the supraspinatus and infraspinatus muscles. As a result, even with a perfectly executed transfer, the baseline capacity for regeneration and the function of the supraspinatus muscle are reduced, leading to impaired abduction function [20]. Combining transfers to both the suprascapular and axillary nerves leads to even better results, because it additionally causes regeneration of the deltoid muscle, which supports the supraspinatus muscle in abduction to a level of at least 90 degrees [17,18,19]. The posterior approach, using the long head of the triceps to transfer to the axillary nerve, is preferred as it avoids misdirection of regenerated axons and minimizes functional loss. The functional loss following the transfer of the long head of the triceps is minimal, as the remaining two heads of the triceps, along with the teres muscle group, compensate for any deficit. Among the three heads of the triceps, the long head plays the least significant role in elbow extension [21,22].

In their work, Bertelli and Ghizoni [20] evaluated the functional outcomes in 110 patients with complete brachial plexus injuries who underwent accessory nerve transfer to the suprascapular nerve within less than a year after injury, with a minimum follow-up period of 2 years. They created two groups of patients based on the surgical approach used—classic (group 1) and extended (group 2). The average range of abduction was 58° (45° in group 1 and 62° in group 2). Active external rotation was present in only 2 out of 29 patients in group 1 and in 32 out of 81 patients in group 2, with an average of 87°. However, this was measured not from the neutral position but from full internal rotation (with the patient’s forearm starting from resting on their abdomen). According to the authors, the significant difference between the two groups was attributed to less dissection and consequently less devascularization of the accessory nerve, as well as the detection of extended lesions in the suprascapular nerve, which allowed for coaptation of the accessory nerve with a healthy distal stump of the suprascapular nerve using an extended approach.

Bhatia et al. [23] reported comparable findings in their study on spinal accessory to suprascapular nerve transfers involving 49 patients with complete brachial plexus palsy. They noted partial restoration of shoulder abduction in 43 of the 49 patients (88%). Specifically, 36 patients regained abduction between 30° and 45°, while 7 patients achieved a range of 70° to 80°.

Leechavengvong et al. [24] documented outcomes from a study involving seven patients with C5 and C6 root avulsion injuries who received double nerve transfers. These transfers utilized the distal spinal accessory nerve for the suprascapular nerve and the long head triceps branch for the anterior branch of the axillary nerve. The average shoulder abduction attained was 124°. In a subsequent study [25], the same team analyzed 15 patients, of whom 8 achieved shoulder abduction ranging from 130° to 160°. In all instances, root avulsions were confirmed before proceeding with surgical reconstruction.

Estrella et al. [26] conducted a comparative study on shoulder abduction and external rotation outcomes in patients with brachial plexus injuries. The study included 20 patients, with 5 undergoing double nerve transfers (spinal accessory nerve to the suprascapular nerve and lateral branch of the triceps or intercostal nerves 3 and 4 to the axillary nerve) and 15 receiving single nerve transfers (spinal accessory nerve to the suprascapular nerve) to restore shoulder function. In the single nerve transfer group, the average shoulder abduction was 71.3 degrees, and external rotation averaged 56 degrees. In contrast, the double nerve transfer group showed significantly better results, with a mean abduction of 123 degrees and external rotation of 86 degrees. The improvement in shoulder abduction was statistically significant, while the difference in external rotation was not. Furthermore, they found no significant difference in shoulder function whether the surgery was performed within 6 months post-injury or later, provided it was performed within 12 months.

In our group of patients, we performed single or double nerve transfers in the shoulder region in 13 patients, achieving an average flexion of 103 degrees, abduction of 83 degrees, and external rotation of 30 degrees. In two patients, no active movement was achieved, and in one more, we observed a weak outcome with 30 degrees of flexion and abduction. However, five patients attained full or nearly full range of motion. We performed double transfers on eight patients, and the average results were comparable to combined results. Surprisingly, the group of patients who underwent a single nerve transfer in the shoulder (five patients) demonstrated the same average range of motion as patients after double nerve transfers. When analyzing each case individually, we found that two patients who underwent a single transfer of the long head triceps branch for the axillary nerve also had a suprascapular nerve neurolysis performed due to a good intraoperative response following nerve stimulation. This initially provided a better prognosis for nerve regeneration and shoulder function, with an average range of motion of 145 degrees of flexion and 125 degrees of abduction, compared to the group of patients with a single nerve transfer and an average of 73 degrees of flexion and 56 degrees of abduction. These results are consistent with the findings of Estrella [26], who reported that double transfers provide a better range of motion in long-term follow-up compared to single transfers. It is also worth noting that the shoulder range of motion in our group of patients is better than that presented by Bertelli and Bhatia [20,23], likely because the majority of our patients underwent either double nerve transfers or single transfers with a favorable prognosis for suprascapular nerve regeneration. Our results are comparable to those reported by Estrella [26] for patients with single nerve transfers, though slightly weaker than those achieved with double transfers or the results reported by Leechavengvong [21,25]. In comparison to Estrella’s study [26], the time between injury and surgery significantly impacted the range of motion outcome in our long-term assessment. Patients who underwent surgery within 6 months of the injury achieved a better range of motion than those operated on after 6 months (but no later than 12 months), with flexion and abduction ranges of 106 degrees and 95 degrees, respectively, compared to 96 degrees and 65 degrees. The influence on the outcome may be due to the fact that patients operated on within 6 months of the injury were younger, with an average age of 23 years compared to 32 years for those operated on after 6 months.

The average range of motion in external rotation did not significantly differ based on the evaluated parameters, such as single or double transfer or the time from injury to surgery.

Elbow flexion could be achieved using various donor nerves, including the medial pectoral nerve [27], the intercostal nerves [28], the thoracodorsal nerve [29], the spinal accessory nerve [30], or the phrenic nerve [31]. Recent techniques such as the transfer of a single fascicle from the ulnar or median nerve to the motor branches of the biceps and brachialis have shown promising results [32,33]. These techniques do not compromise donor nerve function and lead to faster recovery since the transfer is performed closer to the target muscle [32,33].

Liverneaux et al. [34] reported a series of 15 patients who underwent double fascicular nerve transfers to restore elbow flexion, with an average surgery delay of 6.6 months but no more than 12 months. Of the 10 patients who had at least 6 months of postoperative follow-up, all regained at least grade 4 motor strength for elbow flexion, as assessed by the MRC scale. Additionally, no significant motor or sensory deficits were observed in the distribution of the median or ulnar nerves. Among the patients, six had C5 and C6 injuries, three had C5, C6, and C7 palsies, and one suffered from an infraclavicular injury. Mackinnon et al. [35] reported similar findings in their series involving six patients who also underwent double fascicular nerve transfers. After an average follow-up of 20.5 months (at least 13 months), all patients achieved at least MRC grade 4 for elbow flexion. In four of these patients, elbow flexion strength reached MRC grade 4+, while the remaining two achieved grade 4.

Recently, Lee et al. [36] published a systematic review with a meta-analysis that assessed the effectiveness of different nerve transfers in restoring elbow flexion in adults following brachial plexus injury when neurotization was performed within 1 year of injury. They evaluated the outcomes of 12 studies with at least 24 months of follow-up, in which patients with partial brachial plexus injuries underwent double fascicular nerve transfers (double Oberlin) to restore elbow flexion, where 89% of operated patients achieved movement with a strength of M4 or greater. Worse outcomes were observed in patients who underwent a single Oberlin transfer using fascicles of the ulnar or median nerve, with patients achieving M4 or greater strength in 76% and 48% of cases, respectively. The best results were obtained with transfers using two intercostal nerves, as 94% of patients achieved M4 or greater strength, which is comparable to the outcomes following a double Oberlin transfer.

In our group, we performed nerve transfers to restore elbow flexion and strength in 11 patients, achieving an outcome of M4 or greater in 7 patients (64%), while 3 patients attained M3 or M3+ strength, and 1 patient achieved M1. All patients underwent the procedure within one year of their brachial plexus injury. Among the patients who underwent double fascicular nerve transfers, the results were significantly better, with 75% of patients achieving M4 or greater, which shows better outcomes when compared to Liverneaux’s study (67%) [34]. However, in the group of patients who underwent a single Oberlin transfer, only one out of three patients who had a fascicle of the ulnar nerve transferred to the motor branch of the brachialis achieved M4 strength. Furthermore, in this patient, a double transfer was not performed due to an intraoperative favorable response to nerve stimulation, and therefore, neurolysis of the motor branch to the biceps brachii was carried out instead. This offers a better prognosis initially and could be somewhat categorized in the double Oberlin group. Our results after double Oberlin transfers are slightly worse (75% M4 or more) than those reported by Mackinnon (100%) [35] and in Lee et al.’s meta-analysis (89%) [36], although they align in that a higher percentage of patients with M4 or greater outcomes is achieved following double Oberlin transfers compared to single transfers. In both the studies mentioned and in ours, no permanent deficits were observed in the median or ulnar nerves; however, one of our patients experienced temporary sensory weakness in the ulnar nerve distribution. Similar transient sensory disturbances or paresthesia were noted by Bhandari et al. [16] in their work on double Oberlin transfers, lasting 2–3 weeks and affecting either the small finger or the index finger.

## 5. Conclusions

Nerve transfers provide predictable outcomes within a relatively short period of time and are often used in challenging cases, such as avulsion injuries of the spinal nerve roots. The surgical technique is demanding, requiring careful planning of the stages over time. Maximum effects are typically observed after 1–2 years. On average, patients achieve around 100° of flexion and 80° of abduction in the shoulder, with a strength of M3–M4, and around 90° of elbow flexion with a strength of M4 or greater. In both the shoulder and the elbow, double nerve transfers—such as the spinal accessory nerve to the suprascapular nerve and the radial nerve branch to the long or medial head of the triceps brachii to the axillary nerve, or the transfer of motor fascicles of the ulnar and median nerves (double Oberlin) to the brachialis and biceps brachii motor nerves—yield better long-term outcomes than single transfers.

In some cases, certain transfers may be ineffective, and secondary surgeries involving tendon transfers are required.

## Figures and Tables

**Table 1:** Information on all patients with partial brachial plexus injury.

Patient	Sex	Injury	Age at Surgery	Nerve Surgery 1	Nerve Surgery 2	Secondary Procedures	Time from Injury to First Surgery (Months)
1	M	motocycle	18	SSN neurolysis LRN to AxN		Latissimus dorsi transfer	9
2	M	motocycle	31	plexus neurolysis AN to SSN	MB to BB		6
3	M	motocycle	38	AN to SSN RN to AxN	MB to BB UB to BrB	Steindler procedure	5
4	F	bike	25	SSN neurolysis MRN to AxN			5
5	F	motocycle	19	AN to SSN MRN to AxN	MB to BB UB to BrB		6
6	M	motocycle	38	plexus neurolysis	AN to SSN MRN to AxN		6
7	M	fall from height	29	AN to SSN LRN to AxN	MB to BB UB to BrB		5
8	M	motocycle	17	plexus neurolysis AN to SSN	MB to BB UB to BrB	Steindler procedure, trapezius transfer	5
9	M	sport	39	plexus neurolysis	MB to BB UB to BrB		4
10	M	accident	15	AN to SSN RN to AxN			6
11	M	motocycle	48	UB to BrB, neurolysis biceps branch			11
12	M	fall from height	32	AxN neurolysis	MRN to AxN		12
13	M	accident	23	AN to SSN, MRN to AxN	MB to BB UB to BrB		8
14	M	accident	48	AN to SSN, MRN to AxN MB to BB UB to BrB			9
15	F	bike	15	AN to SSN, MRN to AxN	MB to BB UB to BrB		2
16	M	cut	28	UB to BrB			8
sum or average	M-13 F-3		28.9				6.7

M—male; F—female; SSN—suprascapular nerve; AN—accessory nerve; AxN—axillary nerve; LRN/MRN—radial nerve branch to the long/medial head of triceps brachii; UB/MB—ulnar/median nerve motor fascicle; BB—biceps brachii; BrB—brachialis.

**Table 2:** Detailed information on the types of procedures performed, categorized by shoulder and elbow.

	Type of Procedure	Number of Patients
Shoulder	AN to SSN and RN to AxN	8
	SSN neurolysis and RN to AxN	2
	AN to SSN	2
	RN to AxN	1
	plexus neurolysis *	3
Elbow	MB to BB and UB to BrB	8
	MB to BB	1
	UB to BrB **	2

* Additional procedure as a part of nerve transfer or before it; ** one with BB branch neurolysis; SSN—suprascapular nerve; AN—accessory nerve; AxN—axillary nerve; LRN/MRN—radial nerve branch to the long/medial head of triceps brachii; UB/MB—ulnar/median nerve motor fascicle; BB—biceps brachii; BrB—brachialis.

**Table 3:** Range of motion and muscle strength scores in patients after nerve transfers in the shoulder and elbow.

Patient	Range of Motion [°]		Muscle Strenght [MRC Scale]		Follow-Up Time [Months]
	Shoulder [FLEX/ABD/ER]	Elbow [FLEX]	Shoulder [FLEX and ABD]	Elbow [FLEX]	
1	120/80/40	n/a	M3+ *	n/a	24
2	30/30/0	90	M3-	M3	72
3	110/90/40	120	M4	M4+ *	57
4	170/170/30	n/a	M4	n/a	70
5	0/0/0	130	M1	M3+	12
6	170/100/−40	n/a	M3	n/a	96
7	180/180/70	140	M4+	M5	78
8	70/40/40	120	M4 *	M4 *	30
9	n/a	140	n/a	M5	31
10	180/180/50	n/a	M4+	n/a	30
11	n/a	120	n/a	M4	12
12	120/100/30	n/a	M4	n/a	24
13	70/45/60	120	M4	M4	17
14	0/0/0	0	M1	M1	20
15	110/70/45	120	M3+	M4	24
16	n/a	90	n/a	M3+	14
average	103/83/30	97	≥M4:7 M3:4 M1:2	≥M4:7 M3:3 M1:1	38

FLEX—flexion; ABD—abduction; ER—external rotation; MRC—Medical Research Council Scale; n/a—not applicable; *—results after additional tendon transfer.

## Data Availability

Data are contained within the article.
